# Association of maternal leukocyte, monocyte, and neutrophil counts with hypertensive disorders of pregnancy: the Japan Environment and Children’s Study (JECS)

**DOI:** 10.1038/s41598-024-55623-3

**Published:** 2024-03-27

**Authors:** Shiori Ishiyama, Kazuki Mochizuki, Ryoji Shinohara, Kunio Miyake, Megumi Kushima, Reiji Kojima, Sayaka Horiuchi, Sanae Otawa, Hideki Yui, Tadao Ooka, Yuka Akiyama, Hiroshi Yokomichi, Zentaro Yamagata, The Japan Environment and Children’s Study Group, Michihiro Kamijima, Shin Yamazaki, Yukihiro Ohya, Reiko Kishi, Nobuo Yaegashi, Koichi Hashimoto, Chisato Mori, Shuichi Ito, Zentaro Yamagata, Hidekuni Inadera, Takeo Nakayama, Tomotaka Sobue, Masayuki Shima, Seiji Kageyama, Narufumi Suganuma, Shoichi Ohga, Takahiko Katoh

**Affiliations:** 1 Faculty of Life and Environmental Sciences, University of Yamanashi, 4-4-37, Takeda, Kofu, Yamanashi 400-8510 Japan; 2 Center for Birth Cohort Studies, University of Yamanashi, Chuo, Yamanashi Japan; 3 Department of Health Sciences, University of Yamanashi, 1110 Shimokato, Chuo, Yamanashi 409-3821 Japan; 4 Nagoya City University, Nagoya, Japan; 5 National Institute for Environmental Studies, Tsukuba, Japan; 6 National Center for Child Health and Development, Tokyo, Japan; 7 Hokkaido University, Sapporo, Japan; 8 Tohoku University, Sendai, Japan; 9 Fukushima Medical University, Fukushima, Japan; 10 Chiba University, Chiba, Japan; 11 Yokohama City University, Yokohama, Japan; 12 University of Yamanashi, Chuo, Japan; 13 University of Toyama, Toyama, Japan; 14 Kyoto University, Kyoto, Japan; 15 Osaka University, Suita, Japan; 16 Hyogo Medical University, Nishinomiya, Japan; 17 Tottori University, Yonago, Japan; 18 Kochi University, Nankoku, Japan; 19 Kyushu University, Fukuoka, Japan; 20 Kumamoto University, Kumamoto, Japan

**Keywords:** Biomarkers, Risk factors

## Abstract

Hypertensive disorders of pregnancy (HDP) increase the risk of preterm births and cesarean delivery. This study aimed to investigate whether maternal blood leukocyte, monocyte, or neutrophil counts in the first trimester are related to the development of HDP. Data were collected from the Japan Environment and Children’s Study, a large birth cohort study (n = 38,194) that recruited pregnant women in 15 Regional Centers across Japan (from January 2011 to March 2014). The odds ratios (ORs) for mild/severe HDP according to the cut-off value of leukocyte/neutrophil/monocyte counts by the receiver operating characteristic curve showed high ORs. Furthermore, pregnant women with the highest quartiles of leukocyte and monocyte counts had higher adjusted ORs (aORs) for mild (leukocyte: aOR = 1.27, 95% confidence interval [CI]: 1.02–1.58; monocyte: aOR = 1.30, 95% CI 1.04–1.63) and severe HDP (leukocyte: aOR = 1.51, 95% CI 1.08–2.13; monocyte: aOR = 1.44, 95% CI 1.03–2.01) compared with those with the lowest quartiles of those counts. In addition, pregnant women with the highest neutrophil counts had higher aOR for mild HDP (aOR = 1.26, 95% CI 1.02–1.56) compared with those with the lowest count. In conclusion, high leukocyte and monocyte counts in the first trimester are associated with the development of HDP. Thus, they may be used to predict subsequent HDP.

## Introduction

Hypertensive disorders of pregnancy (HDP) are a pregnancy complication known to increase the risk of preterm births and cesarean delivery^1,2^. A study using data from the Japan Public Health Center-based Prospective Study for the Next Generation, a population-based cohort study that included 46,365 women, reported that among pregnant women with singleton births, those with HDP had a higher risk of neonatal low birth weight (< 1500 g) than appropriate birth weight. These findings suggest that early detection and prevention of HDP is important for reducing the number of low birth weight (LBW) infants born preterm or by cesarean delivery.

However, the prediction of HDP is still underdeveloped. The serum soluble fms-like tyrosine kinase 1 (sFlt-1)/placental growth factor (PLGF) ratio has recently been developed for prediction of PE (gestational hypertension nephropathy), one of the four types of HDP^3^. sFlt-1 is an angiogenesis inhibitor that binds to PLGF, an angiogenic factor produced by the placenta, and competes with Flt-1 to inhibit intracellular signaling and suppress angiogenesis, leading to endothelial damage of maternal and placental vessels^4^. Pregnant women with blood sFlt-1/PIGF level above the cut-off value (> 38) had a higher risk of PE within 4 weeks. In recent years, the possible involvement of leukocytes and inflammation in HDP has been demonstrated: neutrophil counts and concentration of interleukin 6 (IL-6), an inflammatory cytokine, were significantly higher in pregnant women with HDP than in their normotensive counterparts, and IL-6 levels in pregnant women with HDP increased with the progression of HDP severity^5^. In addition, pregnant women with higher pre-pregnancy leukocyte counts had an elevated risk of subsequent HDP (odds ratio [OR], 1.6; 95% confidence interval [CI], 1.1–2.3)^6^. Therefore, it is possible that a leukocyte-based inflammatory index could be used as a predictor of HDP.

Many studies suggest that the number of leukocytes in blood is positively associated with the development of hypertension. In a cohort study of 9383 subjects without hypertension at the time of enrollment, 4606 subjects developed hypertension within the 40-year follow-up^7^. Further, a risk of hypertension with increasing leukocyte count was 1.10 times higher in men and 1.05 times higher in women, concluding that the incidence of hypertension can be predicted by increased blood leukocyte counts^7^. Moreover, the study reported that the risk of hypertension from the lowest (≤ 2.6, reference) to the highest quartiles (> 2.6–3.3, > 3.3–4.1, > 4.1) of neutrophil count was 1.18, 1.28, and 1.22 times higher, respectively, showing that increased neutrophil count (× 10^3^/mm^3^) was associated with the incidence of hypertension among women^7^. However, these studies were not conducted among pregnant women.

With respect to the association between events of pregnancy and birth and leukocyte counts, a cohort study of 33,866 pregnant women reported that a higher blood leukocyte count (> 13,800 × 10^9^/L) in the first trimester was associated with a higher risk of premature delivery (< 37 weeks).^8^ In addition, the study showed that compared with pregnant women with lower blood leukocyte counts at first trimester, those with higher counts had higher rates of fertility treatment (10.3% vs. 0%), cesarean section delivery (22.6% vs. 13.0%), small-for-gestational-age infants (5.0% vs. 2.8%), and LBW infants (13.4% vs. 10.9%)^8^. However, there are few studies on the relationship between leukocyte counts, particularly the subtype counts, and HDP. Thus, this study aimed to investigate whether maternal leukocyte, neutrophil, or monocyte counts in early pregnancy could predict the development of HDP.

## Results

### Participant characteristics

Of the 38,194 pregnant women evaluated in this study, 858 and 370 pregnant women had mild HDP and severe HDP diagnosed at delivery, respectively. Table 1 summarizes the maternal, obstetric, and perinatal characteristics of all participants. Tables S1–S3 show the participants’ characteristics according to quartiles of leukocyte, neutrophil, and monocyte counts in maternal blood at the first trimester.

**Table 1:** Participants characteristics (n = 38,194).

Variables	
Maternal characteristics	
Age (years)	30.7 ± 5.0
BMI (kg/m^2^)	21.7 ± 4.0
Primiparity (%)	41.2
Systolic blood pressure (mmHg)	110.8 ± 14.5
Diastole blood pressure (mmHg)	64.1 ± 12.9
Total leukocyte cell counts (/μL)	8027 ± 1930
Neutrophil counts (/μL)	5963 ± 1673
Monocyte count (/μL)	382 ± 115
Higher education (> junior high school)	69.5
Annual household income (< 600 JPY)	74.6
Alcohol intake (%)	9.7
Smoking (%)	4.8
Obstetric characteristics	
Fertility treatment (%)	6.9
Gestational diabetes﻿ (%)	2.6
Mild HDP﻿ (%)	2.2
Sever HDP﻿ (%)	1.0
Placental abruption﻿ (%)	0.4
Cesarean section﻿ (%)	19.8
Preterm pre-labor rupture of membranes﻿ (%)	8.0
Intrauterine infection﻿ (%)	0.6
Maternal infection﻿ (%)	2.2
Preterm delivery < 37 weeks﻿ (%)	4.5
Perinatal characteristics	
Birth weight (g)	3028 ± 416
< 2500 (g)	7.9
2500–3999 (g)	91.2
> 4000 (g)	0.9
Birth height (cm)	48.8 ± 2.3
Head circumstance (cm)	33.2 ± 1.5
Sex (female; %)	48.7
Gestational age (weeks)	38.8 ± 1.6
Congenital malformation﻿ (%)	6.0

Data are presented as the mean ± SD or percentage.

*BMI* body mass index, *HDP* hypertensive disorders of pregnancy, *JPY* Japanese yen, *SD* standard deviation.

### Prediction of HDP and relationship between HDP occurrence at delivery and counts of leukocytes, neutrophils, and monocytes in maternal blood in the first trimester

**Figure 1:**
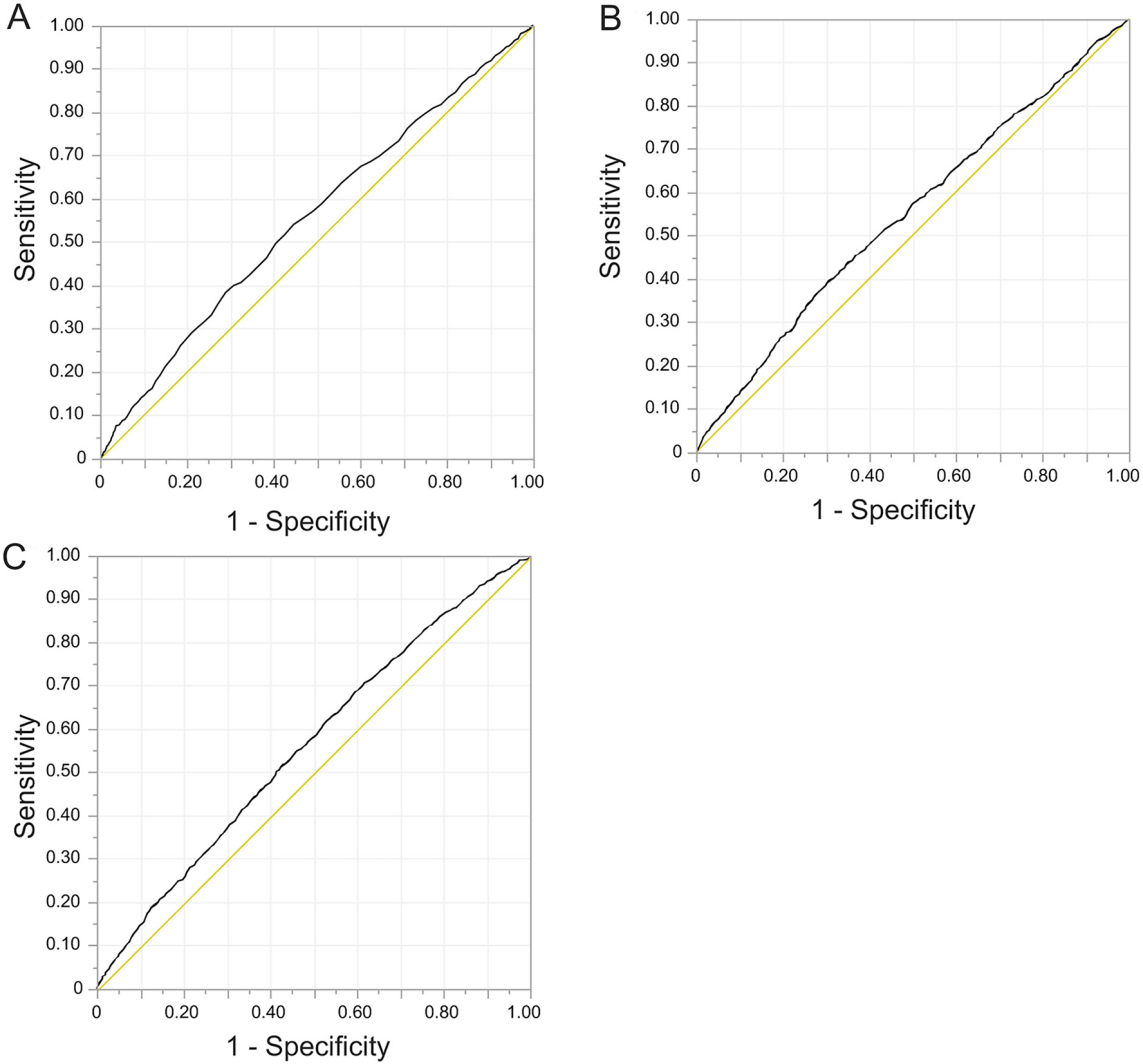
Outcomes of ROC curve analyses for mild HDP. (**A**) Leukocyte counts (cut-off value: 9000, AUC: 55.6%, sensitivity: 38.3%, specificity: 71.1%), (**B**) Neutrophil counts (cut-off value: 6469.5, AUC: 54.9%, sensitivity: 44.1%, specificity: 65.1%), (**C**) Monocyte counts (cut-off value: 336.3, AUC: 56.1%, sensitivity: 70.8%, specificity: 38.2%). *AUC* area under the curve, *HDP* Hypertensive Disorders of Pregnancy, *ROC* receiver operating characteristic.

**Figure 2:**
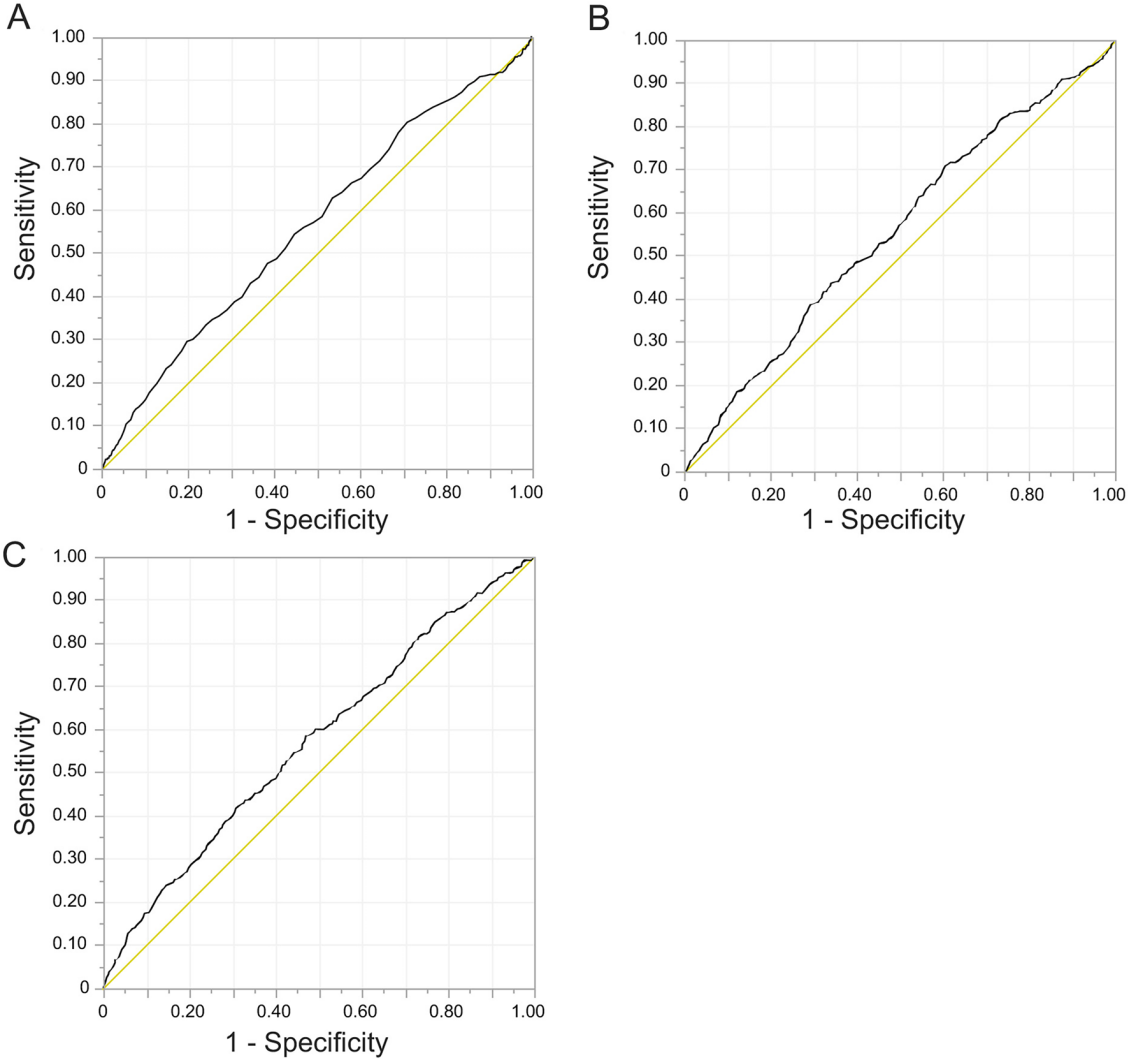
Outcomes of ROC curve analyses for severe HDP. (**A**) Leukocyte counts (cut-off value: 9600, AUC: 56.2%, sensitivity: 29.5%, specificity: 80.2%), (**B**) Neutrophil counts (cut-off value: 5413.1, AUC: 55.5%, sensitivity: 71.6%, specificity: 38.7%), (**C**) Monocyte counts (cut-off value: 376.3, AUC: 57.1%, sensitivity: 58.4%, specificity: 53.0%). *AUC* area under the curve, *HDP* Hypertensive Disorders of Pregnancy, *ROC* receiver operating characteristic.

Receiver operating characteristic (ROC) curve analysis was performed to obtain cut-off values, sensitivities, and specificities (Figs. 1, 2), and the ORs for mild/severe HDP according to the cut-off values were calculated using univariate analysis (Table 2). The groups with counts above the cut-off value had higher ORs for mild HDP (leukocyte; OR = 1.53, 95% CI 1.33–1.76, neutrophil; OR = 1.47, 95% CI 1.28–1.68, monocyte; OR = 1.50, 95% CI 1.29–1.74) and severe HDP (leukocyte; OR = 1.69, 95% CI 1.35–2.11, neutrophil; OR = 1.59, 95% CI 1.27–2.00, monocyte; OR = 1.58, 95% CI 1.29–1.95) compared with groups with counts below the cut-off value (Table 2).

**Table 2:** Odds ratios (95% CI) for mild/severe HDP according to cut-off value among 38,194 pregnancies.

	Maternal leukocyte counts		Maternal neutrophil counts		Maternal monocyte counts	
	Q1 (under cut-off value)	Q2 (over cut-off value)	Q1 (under cut-off value)	Q2 (over cut-off value)	Q1 (under cut-off value)	Q2 (over cut-off value)
Mild HDP	< 9000	9000–<	< 6469.5	6469.5–<	< 336.3	336.3–<
All participants, n	27,060	11,134	24,769	13,425	14,524	23,670
Maternal leukocyte/neutrophil/monocyte counts, means ± SD	7084 ± 1263	10,317 ± 1221	5009.6 ± 1024.9	7723.2 ± 1117.8	275.0 ± 44.6	447.5 ± 93.1
Cases, n	529	329	480	378	251	607
Gestational age at time of blood collection, means ± SD	11.2 ± 2.17	11.3 ± 2.18	11.2 ± 2.16	11.3 ± 2.18	11.2 ± 2.16	11.2 ± 2.18
Crude odds ratio (95% CI)	1.0 (Reference)	1.53 (1.33–1.76)*	1.0 (Reference)	1.47 (1.28–1.68)*	1.0 (Reference)	1.50 (1.29–1.74)*
Severe HDP	< 9600	9600–<	< 5413.1	5413.1–<	< 376.3	376.3–<
All participants, n	30,577	7617	14,752	23,442	20,204	17,990
Maternal leukocyte/neutrophil/monocyte counts, means ± SD	7332 ± 1374	10,817 ± 1174	4385.6 ± 859.5	6956.3 ± 1242.1	297.8 ± 52.9	476.4 ± 88.8
Cases, n	261	109	105	265	154	216
Gestational age at time of blood collection, means ± SD	11.2 ± 2.17	11.3 ± 2.18	11.1 ± 2.15	11.3 ± 2.18	11.2 ± 2.16	11.2 ± 2.18
Crude odds ratio (95% CI)	1.0 (Reference)	1.69 (1.35–2.11)*	1.0 (Reference)	1.59 (1.27–2.00)*	1.0 (Reference)	1.58 (1.29–1.95)*

*BMI* body mass index, *CI* confidence intervals, *HDP* hypertensive disorders of pregnancy, *SD* standard deviation.

*P < 0.05 for logistic regression analysis.

The first quartile, Q1, as the lowest count in each group was used as the reference (Table 3). In Model 1, pregnant women with higher maternal leukocyte counts (Q2/Q4) had an increased adjusted OR (aOR) for mild (Q4; aOR = 1.36, 95% CI 1.11–1.67) and severe HDP (Q2; aOR = 1.48, 95% CI 1.06–2.07 and Q4; aOR = 1.73, 95% CI 1.25–2.38) in the univariate analysis. Pregnant women with higher maternal neutrophil counts (Q2/Q3/Q4) had an increased aOR for mild (Q4; OR = 1.36, 95% CI 1.11–1.66) and severe HDP (Q2; OR = 1.51, 95% CI 1.08–2.09, Q3; OR = 1.40, 95% CI 1.01–1.96, and Q4; OR = 1.51, 95% CI 1.09–2.09). In addition, pregnant women with higher maternal monocyte counts (Q2/Q3/Q4) had an increased aOR for mild (Q2; OR = 1.32, 95% CI 1.07–1.63, Q3; OR = 1.50, 95% CI 1.22–1.85, and Q4; OR = 1.75, 95% CI 1.43–2.14) and severe HDP (Q3; OR = 1.45, 95% CI 1.06–1.99 and Q4; OR = 1.91, 95% CI 1.42–2.58). In Model 2, after adding primiparity and infertility treatment, no distinction was observed between the recalculated and initial leukocyte counts for severe HDP (Q2; aOR = 1.44, 95% CI 1.02–2.02, Q4; aOR = 1.54, 95% CI 1.11–2.13), neutrophil counts for severe HDP (Q2; aOR = 1.50, 95% CI = 1.07–2.09), and monocyte counts for mild (Q1; aOR = 1.28, 95% CI 1.02–1.60, Q2; aOR = 1.37, 95% CI 1.10–1.71) and severe HDP (Q4; aOR = 1.48, 95% CI 1.07–2.04) (Table 3). In Model 3, which considered education level and annual income, no difference was observed between the recalculated and initial leukocyte counts for mild (Q4; aOR = 1.27, 95% CI 1.02–1.58) and severe HDP (Q2; aOR = 1.43, 95% CI 1.01–2.05, Q4; aOR = 1.51, 95% CI 1.08–2.13), neutrophil counts for mild (Q4; aOR = 1.26, 95% CI 1.02–1.56) and severe HDP (Q2; aOR = 1.50, 95% CI 1.06–2.12), and monocyte counts for mild (Q3; aOR = 1.31, 95% CI 1.04–1.64, Q4; aOR = 1.30, 95% CI 1.04–1.63) and severe HDP (Q4; aOR = 1.44, 95% CI 1.03–2.01) (Table 3). Additionally, pregnant women with the highest leukocyte, especially monocyte, counts (Q4) had an increased aOR for severe early-HDP onset (Q4; aOR = 4.51, 95% CI 1.93–10.55) compared to pregnant women with low neutrophil counts (Q1) (Table S4).

**Table 3:** Odds ratios (95% CI) for mild/severe HDP according to quartiles of maternal leukocyte, neutrophil, and monocyte counts among 38,194 pregnancies.

	Quartiles of maternal leukocyte counts				Quartiles of maternal neutrophil counts				Quartiles of maternal monocyte counts			
	Q1 (Low)	Q2	Q3	Q4 (High)	Q1 (Low)	Q2	Q3	Q4 (High)	Q1 (Low)	Q2	Q3	Q4 (High)
	< 6800	6800– < 7900	7900– < 9200	9200 – <	< 4854.4	4854.4– < 5848.2	5848.2– < 6965.475	6965.475– <	< 300.8	300.8– < 369	369– < 447.225	447.225– <
Mild HDP												
Crude odds ratio (95% CI)	1.0 (Reference)	1.08 (0.87–1.33)	1.24 (1.01–1.52)*	1.60 (1.32–1.94)*	1.0 (Reference)	1.03 (0.84–1.27)	1.15 (0.94–1.41)	1.57 (1.30–1.90)*	1.0 (Reference)	1.32 (1.07–1.63)*	1.50 (1.22–1.85)*	1.75 (1.43–2.14)*
Severe HDP												
Crude odds ratio (95% CI)	1.0 (Reference)	1.48 (1.07–2.04)*	1.35 (0.97–1.86)	1.94 (1.44–2.63)*	1.0 (Reference)	1.41 (1.03–1.94)*	1.50 (1.10–2.05)*	1.70 (1.26–2.31)*	1.0 (Reference)	1.23 (0.89–1.70)	1.45 (1.06–1.99)*	1.91 (1.42–2.58)*
Model 1												
Mild HDP												
Adjusted odds ratio (95% CI)	1.0 (Reference)	1.08 (0.87–1.35)	1.19 (0.96–1.47)	1.36 (1.11–1.67)*	1.0 (Reference)	1.01 (0.82–1.26)	1.08 (0.87–1.33)	1.36 (1.11–1.66)*	1.0 (Reference)	1.32 (1.05–1.65)*	1.47 (1.18–1.83)*	1.55 (1.25–1.92)*
Severe HDP												
Adjusted odds ratio (95% CI)	1.0 (Reference)	1.48 (1.06–2.07)*	1.23 (0.88–1.74)	1.73 (1.25–2.38)*	1.0 (Reference)	1.51 (1.08–2.09)*	1.40 (1.01–1.96)*	1.51 (1.09–2.09)*	1.0 (Reference)	1.16 (0.39–1.64)	1.31 (0.94–1.82)	1.69 (1.23–2.31)*
Model 2												
Mild HDP												
Adjusted odds ratio (95% CI)	1.0 (Reference)	1.03 (0.83–1.29)	1.12 (0.90–1.39)	1.21 (0.99–1.50)	1.0 (Reference)	0.96 (0.77–1.20)	1.00 (0.81–1.24)	1.21 (0.99–1.49)	1.0 (Reference)	1.28 (1.02–1.60)*	1.37 (1.10–1.71)*	1.34 (1.08–1.67)*
Severe HDP												
Adjusted odds ratio (95% CI)	1.0 (Reference)	1.44 (1.02–2.02)*	1.19 (0.84–1.68)	1.54 (1.11–2.13)*	1.0 (Reference)	1.50 (1.07–2.09)*	1.27 (0.91–1.78)	1.34 (0.96–1.86)	1.0 (Reference)	1.14 (0.81–1.61)	1.22 (0.87–1.71)	1.48 (1.07–2.04)*
Model 3												
Mild HDP												
Adjusted odds ratio (95% CI)	1.0 (Reference)	1.08 (0.86–1.37)	1.17 (0.93–1.46)	1.27 (1.02–1.58)*	1.0 (Reference)	1.00 (0.80–1.26)	1.06 (0.85–1.32)	1.26 (1.02–1.56)*	1.0 (Reference)	1.24 (0.99–1.57)	1.31 (1.04–1.64)*	1.30 (1.04–1.63)*
Severe HDP												
Adjusted odds ratio (95% CI)	1.0 (Reference)	1.43 (1.01–2.05)*	1.21 (0.84–1.73)	1.51 (1.08–2.13)*	1.0 (Reference)	1.50 (1.06–2.12)*	1.23 (0.86–1.76)	1.37 (0.97–1.93)	1.0 (Reference)	1.12 (0.78–1.60)	1.20 (0.85–1.70)	1.44 (1.03–2.01)*

*BMI* body mass index, *CI* confidence intervals, *HDP* hypertensive disorders of pregnancy, *SD* standard deviation.

Model 1: adjusted for maternal age, pre-pregnancy BMI, maternal age, smoking, drinking, gestational blood pressure (systolic and diastolic), and gestational age at enrollment (weeks). Model 2: adjusted for variables in model 1 in addition to parity and infertility treatment. Model 3: adjusted for variables in model 2 in addition to education and income.

*P < 0.05 for logistic regression analysis.

## Discussion

This study demonstrated that pregnant women with higher leukocyte/neutrophil/ monocyte counts in the first trimester had higher ORs for mild and severe HDP according to each cut-off value than did those with lower counts, indicating its validity as a predictor of maternal leukocyte/neutrophil/monocyte count even without considering any confounding factors. In addition, pregnant women with higher leukocyte/neutrophil/monocyte counts in the first trimester had higher aOR for HDP than did those with lower counts. Collectively, these results suggest the possibility that leukocyte/neutrophil/monocyte counts, particularly monocyte counts, in the first trimester are indicative of subsequent development of HDP.

Inflammatory responses are known to contribute to the development of vascular diseases. Animal and in vitro studies demonstrate that neutrophils and monocytes induce the development of thrombogenesis and atherosclerotic formation by enhancing inflammatory responses, including induction of inflammatory cytokine expression, thrombogenesis, and atherosclerotic formation^9,10^. A case–control study using flow cytometry reported that both non-pregnant women with sepsis and pregnant women with pre-eclampsia had three times higher levels of reactive oxygen species (ROS) in granulocyte and monocyte cells than did healthy pregnant women. Further, the ROS levels in granulocyte cells were 1.5 times higher in pregnant women with pre-eclampsia than in non-pregnant women with sepsis^11^. Another case–control study, which evaluated pregnant women in the third trimester, showed that the levels of myeloperoxidase, expressed on activated neutrophils and monocytes, were higher in the placenta and blood of pregnant with pre-eclampsia than in those without pre-eclampsia^12^. Thus, monocyte and neutrophil counts could be related to the onset of HDP.

In this study, we have demonstrated that maternal leukocytes/neutrophils/monocytes are possible predictors of severe HDP. The serum sFlt-1/PLGF ratio has recently been used as a predictor of HDP, with a focus on PE^3^. sFlt-1/PIGF ratio was reported to predict HDP within 4 weeks^13^. The sensitivity and specificity of sFlt-1/PIGF ratio in 700 Asian pregnant women with suspected PE were 62.0% and 83.9%, respectively. However, the maternal leukocyte, neutrophil, and monocyte counts in our study had lower sensitivity and specificity than those of sFlt-1/PIGF ratio for the development of HDP. Therefore, maternal leukocyte, neutrophil and monocyte counts may be inferior to the sFlt-1/PIGF ratio as predictors of HDP. However, the sFlt-1/PIGF ratio in previous studies was measured in the second trimester to predict HDP in the third trimester within 4 weeks, whereas the leukocyte, neutrophil, and monocyte counts in this study were determined in the first trimester. In the present study, blood monocyte count in the first trimester was positively associated with HDP incidence. Therefore, maternal leukocyte, neutrophil, and monocyte counts may be earlier predictors of HDP diagnosis than sFlt-1/PIGF ratio.

With respect to leukocytes and monocytes, the counts in the first trimester of pregnancy were shown to predict the development of HDP, even when risk factors for increased monocyte count, such as primiparity and infertility treatment, were considered. When stratified by cut-off values calculated from ROC curves, the OR was higher for leukocyte counts than for monocyte counts for both mild and severe HDP. However, the aORs for Models 2 and 3, which were divided by quartiles, showed that monocyte counts were more sensitive to moderate values in the quartiles. Further, while HDP was more common in primiparous than in multiparous women, the risk of HDP-related stillbirth was higher among multiparous women, suggesting more severe HDP in multiparous women^14^. A previous study also reported that the odds of HDP were 1.18 (adjusted OR, 95% CI 1.05–1.33) higher for pregnant women who reported infertility treatment compared to pregnant women who had never received infertility treatment^15^. The mechanisms underlying the association between leukocyte counts, including monocytes, and first delivery, and between leukocyte counts and infertility treatment should be investigated in the future. In addition, a cross-sectional study on maternal peripheral blood mononuclear cells in the third trimester reported that the number of monocytes with HLA-DR antigens, indicating monocyte activation, was higher in pregnant women with preeclampsia than in normal pregnant women, and that the HLA-DR antigen population was positively correlated with the severity of preeclampsia^16^. Combined with the results of this study, maternal monocyte counts are associated with the development of HDP. Future studies should clarify the involvement of monocyte counts or monocyte activation in the development of HDP.

The neutrophil counts were also positively associated with HDP incidence in this study; however, the association disappeared in Model 3, which accounted for educational level and annual income. Therefore, the economic background and educational disparities may influence the association between neutrophil counts and HDP incidence.

In this study, the participants were selected from those who were in their first trimester, and the prevalence of HDP was assessed using data at delivery; therefore, we did not mention the timing (second or third trimester) of HDP onset. However, in many studies including the Japan Environment and Children’s Study (JECS), HDP is classified into two classes, i.e., early-onset (< 34 gestational weeks) and late-onset HDP (> 34 gestational weeks)^17,18^. The JECS demonstrated that multiparas with a higher dietary inflammatory index had aOR of 1.53 for early-onset HDP (95% CI 1.06–2.20) but had aOR of 1.13 for late-onset HDP (95% CI 0.90–1.42) compared with multiparas with a lower dietary inflammatory index before pregnancy^19^, indicating that HDP, especially early-onset HDP, is associated with maternal inflammation. Indeed, we confirmed that pregnancies with the highest maternal monocyte counts had a remarkable increased aOR for severe early-HDP onset, suggesting the possibility that early-HDP onset is attributed to maternal inflammation status exhibited by maternal leukocyte counts, including monocyte counts (Table S4). Taken together, further large longitudinal population data sets are needed to establish how inflammation status before pregnancy or in the first trimester is associated with the timing of HDP onset and how it induces HDP onset.

This study has several limitations. First, the blood pressure levels at enrollment were not measured by the medical staff, and the study only collected self-reported chronic hypertension. However, as only a few participants reported chronic hypertension (0.45%, 170 women), they were not excluded from the study. In the future, it is necessary to evaluate the ROC curves sensitivity, specificity, and associations excluding chronic hypertension by assessed medical staff. Second, because the present study focused on the analysis of predictive factors, the models still contained many confounding factors, such as pre-pregnancy blood pressure, antihypertensive medication status, and pre-pregnancy leukocyte, neutrophil, and monocyte counts, which could not collect in our study. These confounding factors should be considered and compared with leukocyte, neutrophil, and monocyte counts in the first trimester. Third, the present study was conducted among Japanese subjects and in a country with a high rate of older childbearing age. The average childbearing age in Japan is above 32 years, one of the highest among Organisation for Economic Co-operation and Development (OECD) countries, while the average age is below 28 years in the United States^20^. As the proportion of women aged ≥ 35 years in this study was 24.1% (9186 women), this study should be replicated in countries with fewer older births, and racial differences should be evaluated.

The results of this study suggest that increased leukocyte/neutrophil/monocyte counts, particularly leukocyte and monocyte counts, in the first trimester of pregnancy can predict HDP occurrence. This finding has important implications in preventing HDP and ensuring safe delivery and child health.

## Methods

### Study design and participants

**Figure 3:**
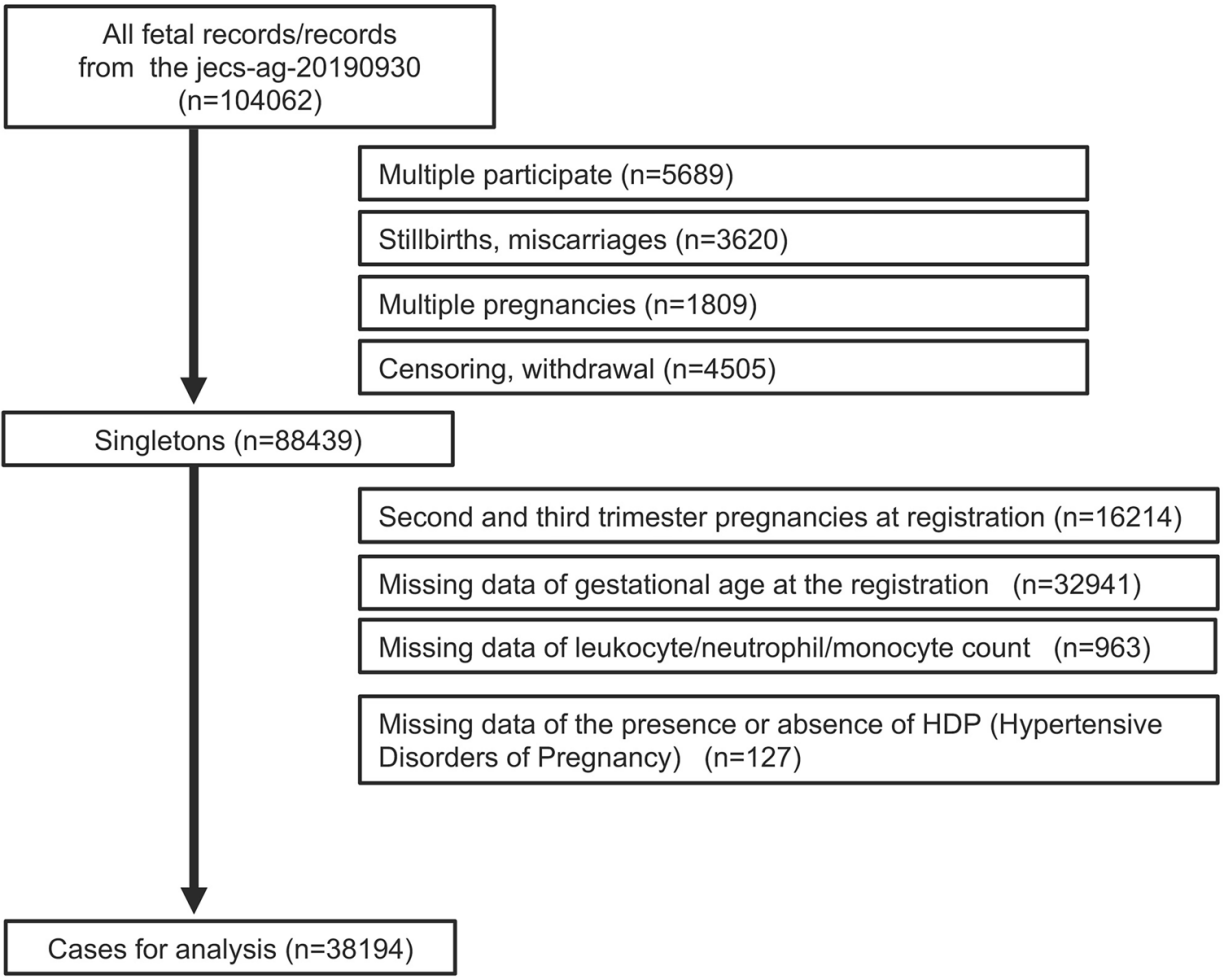
Participant inclusion flow chart.

Data were obtained from the Japan Environment and Children’s Study, a nationwide, government-funded prospective birth cohort study to research the effects of diverse environmental factors on child health and development. The design of this cohort study and protocol has been described previously^21,22^. Briefly, after informed consent was obtained from all participants, 104,062 fetal records in 15 Regional Centers in Japan were registered between January 2011 and March 2014. In this study, the eligibility criteria for the pregnant women were as follows: (1) not in the second and third trimester pregnancies at registration because this study aimed to determine whether maternal blood monocyte or neutrophil counts in the first trimester were related to the development of HDP and (2) complete data of neutrophil count by biochemical test and gestational age at registration. Women with multiple participation, stillbirths and miscarriages, multiple pregnancies, and censored data and who withdrew from the study were excluded. Among the 88,439 pregnant women identified, data of 38,194 eligible mothers with singleton live births were analyzed (Fig. 3).

The JECS protocol was reviewed and approved by the Ministry of the Environment’s Institutional Review Board on Epidemiological Studies and the Ethics Committees of all participating institutions (Ethical Number: No. 100910001), and all of the participants provided written informed consent. The research was conducted in accordance with the Ethical Guidelines for Medical and Health Research Involving Human Subjects established by the Ministry of Education, Culture, Sports, Science and Technology and the Ministry of Health, Labour and Welfare.

### Data collection

The current analysis used the data set released in October 2019 (data set: jecs-ta-20190930), including data from the first trimester, second/third trimester, and delivery. Specifically, we used the following data: (1) collected from a biochemical analysis and included counts of white blood cells, neutrophils, and monocytes at first trimester; (2) collected from a self-reported questionnaire during the second or third trimester and included data of maternal smoking and alcohol status; (3) collected from a clinical outcome record from a medical record transcription that included systolic blood pressure and diastolic blood pressure at the first trimester; (4) collected from a medical record transcription at delivery that included obstetrical outcomes such as gestational age, birth outcome, and obstetric complications; and (5) collected from medical records at registration and included gestational age and maternal age. We obtained data on HDP diagnosis from a medical records transcription^23^. Patients with HDP were diagnosed by a medical doctor based on The Japanese Society of Hypertension in Pregnancy guidelines (JSH2019)^24^, was developed using guidelines of the Japan Society of Obstetrics and Gynecology, and Japan Association of Obstetricians and Gynecologists). In the guideline, HDP is defined as systolic blood pressure ≥ 140 mmHg and/or diastolic blood pressure ≥ 90 mm Hg at gestation without proteinuria and biochemical or hematological abnormalities. Additionally, severe HDP is defined in the guideline as applicable systolic blood pressure ≥ 160 mmHg and/or diastolic blood pressure ≥ 110 mm Hg with preeclampsia, gestational hypertension, superimposed preeclampsia, and chronic hypertension, or exhibition of maternal organ disorder/ uteroplacental dysfunction with/without proteinuria, and mild HDP is defined not severe HDP in the guideline.

### Measurement of maternal leukocyte/neutrophil/monocyte counts

Non-fasting blood samples (33 mL) were collected by medical staff using phlebotomy devices, including needles and vacutainers, provided by the Program Office through its contract laboratory^21^. Of the 33 mL, 9 mL was collected into a vacutainer with coagulant, and the collected samples were transferred to the contract laboratory within 48 h via land or air transportation. The 9 mL sample was allowed to separate into serum and coagulated blood cells. Of the ~ 4 mL of serum, 2 mL was used for clinical chemistry^25^. The leukocyte/neutrophil/monocyte ratio were measured by leukocyte fraction, and the counts were calculated from the ratio.

### Statistical analysis

Participants were divided into four groups according to each quartile of leukocyte/neutrophil/monocyte count (Q1 as the lowest and Q4 as the highest) based on their biochemistry parameters at study registration. Maternal characteristics and obstetric outcomes were then summarized by group. Logistic regression analysis was used to calculate the crude and aORs and their corresponding 95% CIs for mild or severe HDP. The aORs were calculated using three models. Model 1 adjusted for maternal age, body mass index (BMI) before pregnancy, gestational age at registration, gestational blood pressure (systolic and diastolic), maternal smoking status, and maternal drinking status. Model 2 adjusted for the variables in model 1 in addition to parity and infertility treatment. Model 3 adjusted for the variables in model 2 in addition to education level and annual income. Since one of the main objectives of this study was to determine if leukocyte, neutrophil, and monocyte counts could be used as predictors of HDP, a minimum number of confounding factors were selected as covariates for adjustment. BMI was calculated by dividing the maternal weight (kg) by the square of the maternal height (m). Dummy variables, including presence/absence of HDP (0: absence, 1: presence), parity (0: multiparity, 1: primiparity), infertility treatment (0: spontaneous gestation, 1: infertility treatment), drinking status (0: never or quit drink, 1: still drink), smoking status (0: never or quit smoking, 1: 4: still smoke), education level (0: junior high school or less, 1: more than junior high school), annual household income (0: less 600 Japanese yen [JPY], 1: more than 600 JPY), gestational age at registration (1: 0–5, 2: 6–10, 3: 11–15, weeks), maternal age (1: under 19, 2: 20–24, 3: 25–29, 4: 30–34, 5: 35–39, 6: over 40, years), pre-pregnancy BMI (1: 15.5–18.2, 2: 18.3–23.2, 3: 23.3–28.2, 4: 28.3–33.2, 5: over 33.3, kg/m^2^), and gestational blood pressure (systolic; 1: under 99, 2: 100–149, 3: over 150, mmHg, diastolic; 1: under 99, 2: 100–149, 3: over 150, mmHg) were used. ROC analysis was used to confirm if leukocyte, neutrophil, or monocyte count was a valid predictor of HDP. All statistical analyses were performed using JMP Pro version 17 (SAS Institute Inc., Cary, NC, USA). A *p* value < 0.05 indicated statistical significance.

### Details of ethics approval

Ethical Number: No.100910001 (by the Ministry of the Environment’s Institutional Review Board on Epidemiological Studies and the Ethics Committees of all participating institutions).

### Supplementary Information

Supplementary Tables.

## Data Availability

Data are unsuitable for public deposition due to ethical restrictions and legal framework of Japan. It is prohibited by the Act on the Protection of Personal Information (Act No. 57 of 30 May 2003, amendment on 9 September 2015) to publicly deposit the data containing personal information. Ethical Guidelines for Medical and Health Research Involving Human Subjects enforced by the Japan Ministry of Education, Culture, Sports, Science and Technology and the Ministry of Health, Labour and Welfare also restricts the open sharing of the epidemiologic data. All inquiries about access to data should be sent to: jecs-en@nies.go.jp. The person responsible for handling enquiries sent to this e-mail address is Dr Shoji F. Nakayama, JECS Programme Office, National Institute for Environmental Studies. The data that support the findings of this study are available from Dr Shoji F. Nakayama but restrictions apply to the availability of these data, which were used under license for the current study, and so are not publicly available. Data are however available from the authors upon reasonable request and with permission of Dr Shoji F. Nakayama.
